# Social Cognition in Schizophrenia: From Social Stimuli Processing to Social Engagement

**DOI:** 10.3389/fpsyt.2013.00004

**Published:** 2013-02-25

**Authors:** Pablo Billeke, Francisco Aboitiz

**Affiliations:** ^1^Centro Interdisciplinario de Neurociencia, Pontificia Universidad Católica de ChileSantiago, Chile; ^2^Departamento de Psiquiatría, Escuela de Medicina, Pontificia Universidad Católica de ChileSantiago, Chile

**Keywords:** social interaction, interactive view, social loop, game theory, mental state attribution, psychiatry disease

## Abstract

Social cognition consists of several skills which allow us to interact with other humans. These skills include social stimuli processing, drawing inferences about others’ mental states, and engaging in social interactions. In recent years, there has been growing evidence of social cognitive impairments in patients with schizophrenia. Apparently, these impairments are separable from general neurocognitive impairments, such as attention, memory, and executive functioning. Moreover, social cognition seems to be a main determinant of functional outcome and could be used as a guide to elaborate new pharmacological and psychological treatments. However, most of these studies focus on individual mechanisms and observational perspectives; only few of them study schizophrenic patients during interactive situations. We first review evidences of social cognitive impairments both in social stimuli processing and in mental state attribution. We focus on the relationship between these functions and both general cognitive impairments and functional outcome. We next review recent game theory approaches to the study of how social engagement occurs in schizophrenic patients. The advantage of using game theory is that game-oriented tasks can assess social decision making in an interactive everyday situation model. Finally, we review proposed theoretical models used to explain social alterations and their underlying biological mechanisms. Based on interactive studies, we propose a framework which takes into account the dynamic nature of social processes. Thus, understanding social skills as a result of dynamical systems could facilitate the development of both basic research and clinical applications oriented to psychiatric populations.

## Introduction

Schizophrenia is one of the most disabling mental disorders for adults all over the world. In spite of the great advances in antipsychotic medication, people with schizophrenia have poor social integration and several alterations in daily life skills. In recent years, the focus of research in schizophrenia has shifted from reducing classical symptomatology (e.g., positive symptoms) to increasing functionality and social integration (Green and Horan, 2010). In this context, the study of cognitive impairments and social cognitive impairments has become a high-priority area of interest. Social cognition generally refers to the sum of those processes that allow individuals of the same species to interact with one another (Adolphs, 1999; Frith and Frith, 2007). These processes include understanding other people’s emotions, intentions and actions, and acting in social settings. However, social cognition is more than figuring out other people; it also involves *understanding with* others (De Jaegher et al., 2010). Thus, social cognition allows us to sustain interactions, develop relations with others, understand each other, and act together.

Notwithstanding the diversity of the symptoms and presentations of schizophrenia, one of the most impressive common denominators of the illness is a compromised social functioning of the affected individuals (Brüne, 2003). Early descriptions of this pathology include the catatonia, affective symptoms, autism, and the abnormalities in emotional expression and experiences as core features of schizophrenia (Kraepelin, 1899; Bleuler, 1911). Naturalistic behavioral observations in chronic non-medicated patients have revealed that their behavioral repertoire was severely restricted to the defense of a personal “territory,” maintaining a rigid social hierarchy, avoiding any body contact, and the absence of both friendship and mutual help among patients (Staehelin, 1953). Similarly, non-chronic patients with schizophrenia present problems in participating in social settings and developing relations with other people (Grant, 1968). Along this line, Lacan (1973) defines a schizophrenic as a person who specifies himself/herself by not being caught up in any discourse, in any social link. From the philosophical point of view, Deleuze and Gauttari (1972) suggest that schizophrenia may be generated by the conflict between individual desires and behavioral social control (that is, desiring-machines/social-production conflict). Interestingly, these ideas are in line with current evidences which indicate that schizophrenia is related both (1) to alterations in a network of brain areas involved in social processing (Burns, 2004, 2006a,b) and (2) to the evolutionary cost of the changing complexity of post-neolithic society (Abed and Abbas, 2011).

Recently, there has been growing interest in the study of social cognition and its neuro-biological mechanisms in schizophrenic patients. On one hand, clinical research on social skills clearly indicates that this disorder is characterized by substantial, wide-ranging social cognitive impairments (Green et al., 2008). On the other hand, basic neuro-biological studies have identified certain alterations in different brain areas and networks that may underlie these alterations. Nevertheless, there are contradictory findings, likely due to the fact that most of these studies use a first person perspective which follows the classical “perceptual” cognitive paradigm. This way of studying social skills does not capture the wide and rich complexity of the human social world. Interestingly, recent new approaches have challenged this perspective using interactive paradigms to measure social skills in both healthy (De Jaegher et al., 2010) and psychiatric populations (Kishida et al., 2010).

### Domains of social cognitive impairments in schizophrenia

Clinical studies have shown that patients with schizophrenia present alterations in a wide range of psychological tests that measure social skills. Although there is no complete consensus about the hallmark of these impairments, two initiatives have emerged for studying cognitive impairments in schizophrenia that include social domains, namely Measurement and Treatment Research to Improve Cognition in Schizophrenia (MATRICS), and Cognitive Neuroscience for Treatment Research to Improve Cognition in Schizophrenia (CNTRICS; Green et al., 2008; Carter et al., 2009). These initiatives have proposed frameworks to conceptualize social cognitive impairments and psychological tests that measure them. Thus, using these frameworks, it is possible to schematize the social skill domains in the following four areas: emotional processing, social perception, attribution style, and theory of mind (Green et al., 2005; Green and Horan, 2010). Emotional processing involves the capacity to perceive socio-emotional stimuli and the adaptive use of the emotions evoked by them. There is extensive literature indicating alterations in face emotion processing in schizophrenic patients (Marwick and Hall, 2008; Kohler et al., 2010). Social perception refers to the ability to identify roles, rules, and contexts in a social setting. This ability includes not only the perception of individuals acting alone but also the capacity to identify the nature of the relationships between individuals. Attribution style reflects the way people tend to infer the cause of a particular event. For example, psychiatric studies distinguish external personal attributions (causes attributed to other people) from both external situational attributions (causes attributed to situational factors) and internal attributions (causes attributed to oneself). Finally theory of mind, or mentalizing, is the ability to infer, implicitly or explicitly, the intentions, dispositions, and beliefs of others. Theory of mind has been widely addressed in schizophrenia where most of the studies have found consistent impairments when compared with healthy individuals (Brüne, 2005; Sprong et al., 2007).

### Social cognition as an independent domain of general cognition

Why is social cognition studied separately from non-social cognition? For example, in order to figure out other people’s intentions, it is necessary to recruit several processes shared with general non-social cognition, such as attention, memory, and causality detection. Thus, alterations of social abilities could be a consequence of other, more basic impairments of perceptual, or general non-social cognition. Research dealing with this issue distinguishes three lines of evidence which indicate that it is better to understand and study social cognition separately.

One line arises from neuro-biological studies in healthy people, indicating that social tasks activate specific brain areas that can be distinguished from those activated by non-social tasks. Thus, neuroimaging studies show that inferring physical causality activates mainly the lateral prefrontal cortex, whereas social causality tasks activate the medial prefrontal cortex (mPFC; Harris et al., 2005). Similarly, different activation has been observed between inhibitory tasks that involve others’ beliefs (false belief reasoning) and simple (non-social) behavioral inhibition (Rothmayr et al., 2011). Further, a meta-analysis study concludes that if the reasoning is more social, then more activity in the mPFC is generated (Van Overwalle, 2009). Thus, although social cognition requires non-social capacities, the latter does not completely contain the former.

Another line, which is consistent with the above, arises from clinical studies in schizophrenic patients. These studies analyze the relationship between the performance in a series of psychological tests that measure specifically social and non-social skills. Generally, they have shown that social impairments dissociate from non-social domains. For example, using structural equation modeling and factorial analysis, two reports have shown that the variance of the data is better explained when both social and non-social cognition processes are two separate factors (Allen et al., 2007; Sergi et al., 2007). In other words among patients, the variations in the results of cognitive tests are structured in two separate, though related, domains, namely social and non-social cognition.

Other studies have investigated how cognitive impairments impact in the daily functioning of these patients. Daily functioning measures how people perform in everyday situations, including instrumental activities, interpersonal functioning, and vocational achievement. These works reveal that the impairments in daily functioning of schizophrenic patients are related to both social and non-social cognitive domains (Couture et al., 2006, 2011). However, when social and non-social skills are analyzed together, they are better predictors of functional outcome (Couture et al., 2011). This means that social and non-social domains have an independent influence on the everyday performance of these patients. Further, several studies show that social cognition has a mediator effect between neuro-cognition and functional outcome (Couture et al., 2006; Bae et al., 2010; Schmidt et al., 2011b). In other words, social and non-social cognition are associated with functional outcome when they are studied separately, but when they are analyzed together, non-social cognition reduces or loses its association with functional outcome. That is, it is possible that the impact of non-social cognitive impairment in daily functioning occurs through social cognitive impairments. Hence, it has been proposed that social cognition is a more proximal factor in the causal mechanism leading to real world performance. For example, to develop interpersonal relationships, alterations in theory of mind have stronger influence than memory alterations. However, memory impairment can influence theory of mind performance which in turn influences the ability to develop interpersonal relationships.

Finally, the last reason for focusing on social cognition impairments is that they seem to be a life history stable trait that precedes, and even predicts, the illness onset. Using a videotape recording of a cohort of children having lunch, a study showed that alterations of social behavior were the most significant predictors of those children who developed schizophrenia in adulthood, even more so than neuro-motor deficits (Schiffman et al., 2004). Moreover, individuals at ultra-high or family risk for psychosis present social cognition alterations, especially in theory of mind (Chung et al., 2008; Anselmetti et al., 2009; Eack et al., 2010; Gibson et al., 2010; Kim et al., 2011a). These alterations can predict the psychotic conversion (Chung et al., 2008; Anselmetti et al., 2009; Eack et al., 2010; Gibson et al., 2010; Kim et al., 2011a). Consistent results were obtained in a study comparing social skills in prodromal, first episode, and chronic patients, as well as in a longitudinal one of first episode patients (Green et al., 2012; Horan et al., 2012). Together, these studies show that social alterations are a stable trait across the illness.

Taken together the results above, we have that social impairments in schizophrenia are separable from general non-social cognition and have a strong impact in daily performance. Thus, social cognition seems to be a susceptible target of treatment for improving the integration of these patients in society. Indeed, social cognition is a potential prognostic factor for focusing early intervention in susceptible populations. In this context, neuro-biological and translational studies are crucial to the better understanding of the underlying mechanisms of these impairments, which allows us to elaborate adequate interventions. In the subsequent sections, we review the main findings in this area, and we elaborate a framework that can guide future research.

### Functional neuroimaging studies: Methodological issues

In recent years, there has been an exponential increase in functional neuroimaging studies (mostly using functional magnetic resonance imaging, fMRI) and a sustained growth in electroencephalographic (EEG) and magnetoencephalographic (MEG) studies (Smith, 2012). Each of these techniques has advantages and limitations, which must be taken into account before reviewing current evidence.

The EEG is a direct measure of the electric field (or the magnetic field, in the case of the MEG) generated by the electrical activity of the brain at the scalp surface level. In spite of its high temporal resolution (milliseconds), each electrode integrates an area of 10 cm^2^, having a low spatial resolution (Buzsáki et al., 2012). On the other hand, fMRI displays a hemodynamic signal and has a much better spatial resolution than EEG. Specifically, the fMRI measures the blood oxygenation level dependent (BOLD) signals which represent the oxygenated hemoglobin. It is assumed that a stronger BOLD signal reflects a greater demand for oxygenated blood when neurons become electrically active in response to a task (Logothetis, 2008). This change in the oxygenation level takes place after neuronal activity with a peak delay of 4–5 s (Monti et al., 2010; Murayama et al., 2010). This poor temporal resolution of fMRI implies serious limitations for both the methodologies used (e.g., statistics methods; Vul et al., 2009) and for the conclusions of the results. For example, in social interaction studies using imaging techniques, long time response intervals (e.g., larger than 12 s) are frequently used to optimize the BOLD signal. However, in social tasks, changes in timing modify the behavior of players, likely changing the underlying biological processes (Smith and Silberberg, 2010; Rand et al., 2012). Interestingly, new complementary techniques, like high-density EEG recording combined with source-modeling that can account for gyri and sulci (as inferred from MRI imaging) of the subject, and simultaneous EEG-fMRI recordings, can substantially improve the limitations for each methodology considered alone (Esposito et al., 2009; Lei et al., 2011).

## Perceiving Others

**Figure 1:**
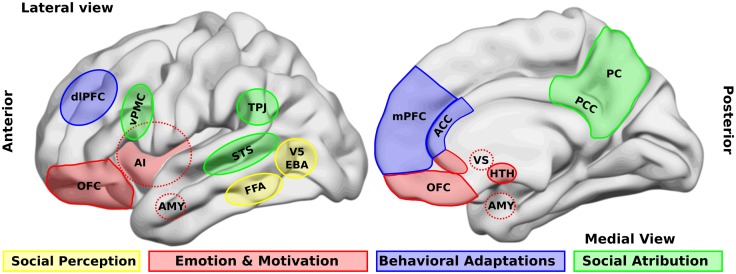
**Brain areas that participate in social processing**. A simple classification of brain areas involved in social processing differentiates regions that participate in four related processes. The first is the perception of basic social stimuli, such as biological motions (V5), part of the body (extra-striate body area, EBA), and faces (fusiform face area, FFA). Another process includes emotional and motivational appraisal, where the amygdala (AMY), the anterior insula (AI), the subgenual and perigenual anterior cingulate cortex (ACC), as well as the orbitofrontal cortex (OFC) participate. These cortical structures are in interaction with subcortical structures as the ventral striatum (VS), and the hypothalamus (HTH). These structures in turn interact with other regions which participate in the goal-directed, adaptive behaviors, and the categorization processes, such as the dorsolateral and the medial prefrontal cortex (dlPFC, mPFC) and the ACC. Finally for social attribution, areas like the ventral premotor cortex (vPMC), the superior temporal sulcus (STS), the AI, the posterior cingulate cortex (PCC), and the precuneus (PC) participate in more automatic, bottom-up inferences of other people’s mental states; whereas structures like the mPFC and the temporo-parietal junction (TPJ) are involved in more cognitive theory of mind skills.

**Figure 2:**
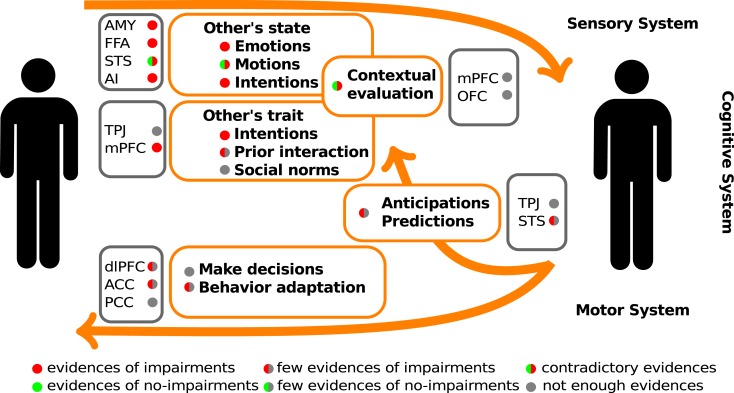
**Schematic summary of the evidences of social cognitive impairments and their underlying neuro-biological mechanisms in schizophrenic patients**. The color circles represent the levels of available evidences from behavioral data (within orange rectangles), and neuroimaging data (within gray rectangle). AMY, amygdala; FFA, fusiform face area; STS, superior temporal sulcus; AI, anterior insula; TPJ, temporo-parietal junction; mPFC, medial prefrontal cortex; dlPFC, dorsolateral prefrontal cortex; OFC, orbitofrontal cortex; ACC, anterior cingulate cortex; PCC, posterior cingulate cortex.

One of the first steps in socio-cognitive processing is the identification of both social relevant stimuli and their emotional value (Ochsner, 2008). Several brain areas, such as the amygdala, the insular cortex, and some regions of the temporal cortex, have been related to this step in healthy people. Two types of stimuli have been the most studied in this context, namely human faces and biological movements. Interestingly, patients with schizophrenia present alterations in the perception of these stimuli. For a schematic representation of the principal brain areas participating in social processing, see Figure 1, and for a schematic summary of the principal evidences in schizophrenic patients, see Figure 2.

### Others’ faces

Undoubtedly, the human face is one of the most important sources of social information. Thus, a great deal of research has been carried out on face processing in both healthy and schizophrenic people. Face identification and face emotion recognition have been two of the most studied and reported impairments in schizophrenic patients (Whittaker et al., 2001; Marwick and Hall, 2008; Kohler et al., 2010; Li et al., 2010). In relation to face recognition, studies generally use tasks where people have to decide whether two faces in different positions are the same person or whether the face presented is a familiar/famous person. These studies mostly show that performance of schizophrenic individuals is worse than that of healthy individuals (Marwick and Hall, 2008). In imaging studies, these impairments are related to functional and structural alterations of the fusiform gyrus (Quintana et al., 2003; Marwick and Hall, 2008). Using EEG, the N170 event-related potential (ERP) has been associated to structural face processing, being likely generated in the fusiform gyrus. Most studies on schizophrenic patients have found a decrease in the amplitude of this component (Campanella et al., 2006; Caharel et al., 2007; Turetsky et al., 2007; Lynn and Salisbury, 2008), although some have found that it is normal or increasing (Wynn et al., 2008; Ramos-Loyo et al., 2009). One possible explanation for the discrepant results is that the deficit depends on the degree of other cognitive demands, mainly memory load (Whittaker et al., 2001). Another explanation is that this deficit depends on early visual processing. In most of the preceding studies, there is a consistency between the modulation of the N170 and that of the P100, which is an early ERP component that reflects early visual processing (Campanella et al., 2006; Caharel et al., 2007; Wynn et al., 2008; Lee et al., 2010). More research is needed to further understand these early stages of structural face processing in schizophrenia.

Another important characteristic of the human face is the transmission of emotional states. The ability to recognize the emotions displayed by others is crucial in social interactions. It has been proposed that misinterpretation of facial affect is a mechanism that can lead to symptoms such as paranoia and persecutory delusions in schizophrenia (Frith, 1996). Despite heterogenic designs and paradigms used to address this issue, a recent meta-analysis of 86 studies reveals a large deficit in emotional face processing in schizophrenic patients (Kohler et al., 2010). In this work, the educational factor, as a rough measure of neuro-cognition, was not related to emotion recognition. This finding agrees with another report which asserts that affect recognition is an independent factor of non-social cognition (Pan et al., 2009). Interestingly, early visual processing and face identification influence the impairments in affect recognition only for negative emotions, but not for positive emotions (Norton et al., 2009). This fact suggests a close relationship between affect recognition and visual processing, but the former is not fully explained by the latter. Similarly, social cognition mediates the effect of early visual alterations in the functional outcome of schizophrenic patients (Sergi et al., 2006). Notably, when the patients had evaluated faces with either contextual sentences or concomitant emotional congruent sounds, their performances were similar to those of healthy people (Lee et al., 2012; Müller et al., 2012). These results stress the need for developing more ecological experimental tasks in order to extrapolate the laboratory data to the daily performance of patients.

Concerning the neurobiology of this impairment, when processing facial expressions of emotions, neuroimaging studies have shown that schizophrenic patients activate regions similar to those of control samples, namely bilateral amygdala and right fusiform gyrus (Li et al., 2010; see Figure 1). Most of these studies have found that the extent of the activations in these patients is more limited, while other studies revealed that schizophrenic patients over-activated the amygdala when observing neutral faces. This effect could produce the observed hypo-activation while looking at fearful faces (Kosaka et al., 2002; Holt et al., 2006; Surguladze et al., 2006; Hall et al., 2008). Interestingly, a patient with amygdala damage recovered the ability to recognize fearful faces when she was instructed to fix her attention on the eyes (Adolphs et al., 2005), and other studies have shown that only early amygdala damage generates theory of mind impairment in contrast to adulthood damage (Shaw et al., 2004). The preceding facts indicate that the amygdala may have a role in some precursor processes of social cognition, such as joint attention. When comparing these findings with those of schizophrenic patients, some authors have proposed a dysregulation of the amygdala in response to social stimuli in schizophrenia. This dysregulation is probably generated by an impaired control from the mPFC (Brunet-Gouet and Decety, 2006).

Another brain area related to emotion recognition impairment in schizophrenic patients is the insular cortex. The insula is an interoceptive cortex which senses the inner state of the body. Its alterations have been reported in many diseases, such as anxiety disorders and drug addiction (Contreras et al., 2008, 2012). A specific role of the insula in the recognition of emotional expressions of disgust, a primitive function related to the appraisal of distasteful or dangerous stimuli, has been reported (Phillips et al., 1997; Wicker et al., 2003). In schizophrenic patients, a diminished activation of the anterior insula has been associated to a deficit in recognizing the expression of disgust (Phillips et al., 1999; Williams et al., 2007). However, the biological mechanism underlying this alteration is not simple. Using neuro-feedback to increase the anterior insula activity in schizophrenic patients, a recent study showed an improvement in disgust recognition, while a worsening in happy face recognition (Ruiz et al., 2013). This result highlights the participation of a network of interacting brain areas instead of specialized, isolated areas.

On the other hand, EEG studies have focused on the N250 component, which is modulated by the emotional valences of faces (Krolak-Salmon et al., 2001). This component presents a fronto-central topography and has been related to a later emotional processing of faces. In schizophrenic patients studies have found mixed results. Two studies found a normal N170, although reduced N250, components, suggesting that deficits in facial affect recognition are not due to abnormalities in facial feature encoding, but rather due to specific deficits in the decoding of emotional information (Streit et al., 2001; Wynn et al., 2008). Other studies found abnormal N170, though normal N250, components, implying that facial feature encoding is abnormal, but affect decoding remains unaffected (Streit et al., 2001; Johnston et al., 2005). Interestingly, when examining the results of the preceding studies, P100 alterations are in concomitance with N170 alterations, though not with N250 alterations. In schizophrenic patients, structural face processing is probably more dependent on early visual processing, whereas emotional processing is more dependent on other alterations. Thus, patient-to-patient variations could lead to the observed differences in the results. Similarly, a behavioral study revealed that impairments in both early visual processing and structural face processing do not explain the affect recognition impairment (Norton et al., 2009). Nonetheless, there have not been enough studies carried out to corroborate this possibility.

### Others’ movements

Another important social stimulus is the movement of social agents. Humans have the tendency to interpret motion as social and intentional if it has biological plausibility despite the fact that its agent may not be biological. For example, geometric shapes moving on a screen (Heider and Simmel, 1944). Moreover, the biological coherent motion of light points representing joints is interpreted as human movements (Johansson, 1973). There are several studies which reveal that the perception of biological motion is related to an action-observation network (or mirror network), which is generally considered to consist of three bilateral cortical areas that are reciprocally connected, namely the ventral premotor cortex, the inferior parietal lobule, and the superior temporal sulcus (STS, see Figures 1 and 2; Keysers and Perrett, 2004). Interestingly, biological motion perception has been proposed as a hallmark of social cognition impairments in developmental diseases, like autism (Pavlova, 2012). There are, however, few studies about perception of biological motion in schizophrenic patients. Studies in this step of social processing are important, since biological motion perception activates several areas involved in schizophrenic symptomatology. For example, the STS and the inferior-parietal lobule (including the temporo-parietal junction, TPJ, Figures 1 and 2) are implicated in mental state attribution (MSA), emotion, and self-representation or agency. Further, these regions are either adjacent to or overlapping with regions implicated in language-related symptoms of the schizophrenic syndrome, such as auditory verbal hallucinations (Torrey, 2007; Wible et al., 2009). Thus, research on this step of social processing can provide more evidences of the physiopathology chain leading to schizophrenic symptoms.

In this context, reports about biological motion using light points show that schizophrenic patients have an altered perception as compared to healthy individuals (Kim et al., 2005, 2011b; Singh et al., 2011). In EEG studies, this impairment is related to a decrease of the μ rhythm suppression that is related to the mirror activity of sensory-motor cortex (Singh et al., 2011). In neuroimaging studies, the impairment has been associated to an alteration of the STS activity (Kim et al., 2011b). Behaviorally, schizophrenic patients tend to describe non-biological motion as biological. Similarly, they present a greater STS activity in non-biological motion than that of control samples (Kim et al., 2011b). Interestingly, there is no difference between the activities of visual area MT (related to motion perception) of both groups. This finding reveals that the impairment of biological motion perception is not due to basic visual alterations. There could probably be either an STS dysregulation or a decoupling of STS activity from other areas of the social brain network. Similarly, studies of emotion processing show a dysregulation in the amygdala activity. However, in light of current evidences, this is only a speculative interpretation requiring further analyses.

In summary, patients with schizophrenia present alterations of the perception of specific social stimuli, such as face emotion and biological motions. These alterations are not fully explained through either early perceptual or non-social cognitive impairments. Temporal regions, like the amygdala and the STS, participate in the neuro-biological mechanism underlying these alterations, which is best described as activity abnormally evoked by non-social stimuli. Interestingly, this is consistent with interpretations which point out that over-interpreting stimuli as social and intentional is the basis for paranoid symptoms and persecutory delusions (Abu-Akel and Bailey, 2000).

## Understanding Others

In order to participate in the rich human social life, it is necessary not only to perceive others, but also to understand them. Crucially, we attribute an inner mental world to those agents, and we infer their intentions, beliefs, and wishes through several sources. The ability to do so, called MSA, or theory of mind, or mentalizing, allows us to maintain social interactions. In healthy people, neuro-biological studies typically contrast mentalizing tasks with control conditions in which no MSA is required. These studies have identified the activation of a network of brain areas which includes the mPFC, the STS, the TPJ, and the inferior parietal lobule (Van Overwalle, 2009). Functional and structural disruption of the neuro-biological mechanisms underlying the MSA may give rise to various mental illnesses that include schizophrenia (Frith, 2004).

There are several evidences indicating an MSA impairment in schizophrenic patients (Brüne, 2005; Sprong et al., 2007). Interestingly, this deficit is independent of general cognitive impairments, such as attention, memory, and executive function. For example, when it is necessary to infer others’ mental states, patients sorting a sequence of pictures do not perform as well as when it is necessary only to infer physical causality (Brunet et al., 2003a). Regarding neuroimaging studies, the most replicated finding is a reduction of mPFC activity as compared to that of control subjects (Brunet-Gouet and Decety, 2006). This pattern has been replicated using diverse paradigms, such as short history comprehension and comic-trip protocols. By contrast, research on mentalizing has led to contradictory results concerning temporal and parietal regions. Using non-verbal comic-trip tasks, studies have found a hypo-activation of the TPJ (Benedetti et al., 2009; Walter et al., 2009; Vistoli et al., 2011b), whereas others have found a hyper-activation of the TPJ and the STS (Brüne et al., 2008; Benedetti et al., 2009). Moreover, a study has found strong STS activity in both schizophrenic patients and control individuals (Brunet et al., 2003b). Interestingly, studies using MEG and EEG in healthy people have shown that parietal and temporal activations occur earlier than frontal activation (McCleery et al., 2011; Vistoli et al., 2011a; Billeke et al., 2012). For example, in an interactive game, there is an activity in the alpha range which anticipates the other player’s behavior. This activity is first found in the temporo-parietal region and, subsequently, in the medial prefrontal region (Billeke et al., 2012). Using MEG in schizophrenic patients, the STS, the TPJ, and the inferior parietal lobule show a hypo-activation which correlates with task performance (Vistoli et al., 2011b). One possible explanation for these disparate results is the existence of an abnormal agency processing in schizophrenia (Brunet-Gouet et al., 2011). The hyper-activation of the parietal regions and the cingulate cortex has been related to passivity symptoms (Spence et al., 1997). Thus, the increase of parietal activity related to mentalizing tasks could be due to the tendency to perceive one’s own thoughts and actions as alien-made (Brüne et al., 2008).

However, the relationship between the finding in the MSA task and functional outcome is not straightforward. In most of the MSA tasks, people have to evaluate social situations in which they do not participate. Thus, the classical MSA paradigms require the ability to take a perspective out of the social exchange that is being assessed. Therefore, on the one hand, performance in the classical MSA task might be affected by the impairments in the capacity to take a perspective or agency. On the other hand, in everyday situations, people require the capacity to attribute intentions to their partners during a social exchange. These “online” MSAs are not assessed in classical tasks (Frith and Singer, 2008). In our next section, we review recent evidences using interactive paradigms to evaluate “online” social processing.

## Understanding with Others

The goal of the social skills of above is to allow us to participate adequately in social exchanges. In order to do so, however, we require to not only perceive social agents and understand their inner worlds, but also need the capacity to make decisions, anticipate their decisions, and act together. This is a crucial step in the chain of social cognitive abilities that lead us to surf properly in our complex social world. Despite this fact, only recently has cognitive neuroscience begun to study social skills in interactive settings where subjects have to make decisions in accordance with how they believe their partners might react. For example, there is growing interest in studying how people coordinate their motor actions in social settings (Sebanz et al., 2006; Dumas et al., 2010; Schmidt et al., 2011a). Using game theory, recent research has assessed the way people interact with each other and how they compete or cooperate for an incentive (Camerer and Fehr, 2006; Lee, 2008). The game theory approach has a series of advantages over classical “perceptual” paradigms that enables us to evaluate social cognition in mental diseases (Kishida et al., 2010) as follows. First, these paradigms give us the possibility to evaluate social decision making in ecological paradigms where the result of a player’s decision depends on the decisions of the other player. Next, game theory provides a series of “optimal” decisions or behaviors in each game. This optimal, or normal, behavior is a function of both the rational construction of the game and the social norm of fairness. Interestingly, this social norm varies according to cultural differences (Henrich et al., 2005, 2006, 2010). Therefore, optimal behavior represents an indicator of social adaptation. Finally, we can evaluate how the behavior of a specific population, for example patients with schizophrenia, deviates from the optimal solution in a given setting. Importantly, since this deviation can be measured and correlated with certain biological activity, it could help us to identify biological markers of this behavior and potential targets of medical intervention (Kishida et al., 2010).

Using game theory, research on schizophrenic patients could shed light on their ability to maintain social exchange. Recently, there has been a series of studies on schizophrenic patients playing the ultimatum game (Agay et al., 2008; Wischniewski et al., 2009; Csukly et al., 2011; van’t Wout and Sanfey, 2011; Wischniewski and Brune, 2011). This game involves two players, the proposer and the responder, who have to split a certain amount of money following a bargaining logic of “take-it-or-leave-it” (Güth et al., 1982). The proposer makes an offer as to how the money should be split between the two. The responder can either accept or reject the offer. If the offer is accepted, the money is split as proposed, but if it is rejected, neither player receives any money. Rationally, the proposer should offer the least sum of money other than zero, and the responder should accept any non-zero offers. However, as we remarked above, behaviors in this game also integrate the social norm of “fairness.” Thus, in occidental societies, proposers offer 40% of the money to be split as their modal offer and responders reject offers less than 30% half of the times.

Interestingly, schizophrenic patients deviate from this normal behavior. As proposers, these patients make offers greater than the 50% social norm, called “hyperfair” offers (Agay et al., 2008). Moreover, patient behavior is less strategic. Control individuals tend to increase their offers after a rejection and decrease them after an acceptance, whereas patients make more erratic offers after a rejection. Similarly, using a sample of students, a study reveals that schizotypal traits correlate with the sum of money offered (van’t Wout and Sanfey, 2011). In other words, normal people with schizotypal traits tend to offer more money (“hyperfair” offers) than people without them. This study also used another version of the game, called the dictator game, that is similar to the ultimatum game where the receptor plays a passive role without the possibility of rejecting the proposer’s offer. Notably in this game, schizotypal traits do not correlate with the money offered. This means that, in the ultimatum game, the hyperfair offers are not due to a different understanding of fairness, but due to a strategic behavior. This fact is corroborated by the behavior of the schizophrenic patients playing a third-party punishment game. In this game, the patients, see other people playing the dictator game and have the possibility to punish the dictator for an unfair offer, or in other words they can decide to lose a portion of their earnings in order to punish the dictator. Interestingly, under these conditions, schizophrenic patients punish the dictator’s unfair offers just as healthy people do (Wischniewski and Brune, 2011). On the other hand, as responders, schizophrenic patients present a greater rate of acceptances of unfair offers and a smaller rate of rejections to fair offers (Csukly et al., 2011). However, the rejection rate increases as a function of the unfairness of the offers just like in healthy individuals (Wischniewski and Brune, 2011). The students with schizotypal traits used in the study of above presented the same pattern of behavior (van’t Wout and Sanfey, 2011).

Taken together, these results show that schizophrenic patients deviate from normal behavior by making more fair offers and accepting more unfair offers. This pattern is not explained by the change of the sense of fairness because the patients reject unfair offers and punish the other players’ unfair offers. However, when they face a person who can punish their behavior (rejecting their offer), they make “hyperfair” offers. A possible explanation for these behaviors is that schizophrenic patients can evaluate fairness and anticipate the others’ decisions, but avoid possible punishments by increasing the offer. In a certain way, this is congruent with a study that uses a variation of the public goods game. In this game, each player (commonly more than two) invests a portion of his/her earnings in both private and public accounts. The allocation of the gathered public goods is equal regardless of the amount of each individual’s investment. If a minimum proportion of the participants invest in the public accounts (the minimum public goods), all receive an additional incentive. A free riding is someone who invests no-money in the public accounts, but receives the investment of the other participants. Schizophrenic patients playing this game present a smaller rate of free riding (Chung et al., 2013) which the authors interpret as impairments in the sensitivity to losses. However, this is also consistent with an attitude to avoid a possible social rejection represented, in this case, by the failure to achieve the minimum public goods. Nevertheless, this does not explain the more erratic behavior after either a rejection in the ultimatum game or a failure in the public goods game. The patients probably also have problems in anticipating how their partners could react to their decisions. Thus, an impairment of this pragmatic aspect of the MSA may cause these erratic behaviors after a negative response.

These interactive studies are still incipient and most of them evaluated only one-shot games. However, the daily social situations are characterized by a repetitive interaction with the same people. Thus, the history of previous interactions is crucial to both figuring out the others’ intentions and adapting our decisions accordingly. Our laboratory results show that healthy people take into account prior interactions with the same partner in order to both anticipate their partner’s behaviors and elaborate their reactions to these behaviors. Interestingly, the mPFC and the temporo-parietal region are strongly related to these processes (Billeke et al., 2012). In the context of schizophrenic patients, it is important to begin using interactive and repetitive paradigms with certain biological measurements, such as neuroimaging or EEG, in order to unravel neuro-biological mechanisms underlying social impairments. We believe that new approaches to social cognition, like game theory, will give us the opportunity to achieve this.

## Models for Social Cognition

Recently, several theoretical frameworks have been proposed to guide research on social cognition impairments in schizophrenia. In this context, findings in social and affective neuroscience are relevant to proposing separable psychopathological constructs based on the knowledge of cortical functions.

Thus, Ochsner (2008) proposes several dissociated constructs which participate in socio-emotional processing. This model proposes five constructs based on differentiated brain systems to distinguish dissociated impairments in schizophrenia. The first is the acquisition of social values and responses. The amygdala and the striatum are the two neural systems most strongly implicated in affective learning. These systems are evolutionarily old and receive multi-modal perceptual inputs. The mPFC and the insula also play key roles in affective learning via interconnection with the amygdala and the striatum. The second construct is recognizing and responding to social affective stimuli. Striatal and mPFC systems have also been implicated in recognizing stimuli whose values they encode. Interestingly, as we have shown above, other structures implicated in this construct are the amygdala and the STS which are related to the alteration of the perception of social stimuli in schizophrenic patients. The third construct is the “embodied simulation” or low-level mental state inference. Based on studies of “mirror systems,” several areas participate in this shared representation, including the inferior parietal, the premotor, the cingulate, and the insular cortices. These areas provide a basis for the vicarious empathic experience and, therefore, for the understanding of others’ actions, pains, or disgusts (Frith and Frith, 2012). The fourth construct is the high-level mental state and trait inferences. In this construct, the more complex judgments about others are processed. The participating areas are the mPFC, the STS, the TPJ, and the medial parietal regions. These areas have also been implicated in schizophrenic impairments. The fifth, and final, construct is the context sensitive regulation. This construct concerns the ability to regulate one’s judgments about and behavior toward others in a context appropriate manner. This regulatory ability manifests it self in different ways with distinct sets of underlying neural systems in lateral and medial prefrontal regions.

### Social loop

**Figure 3:**
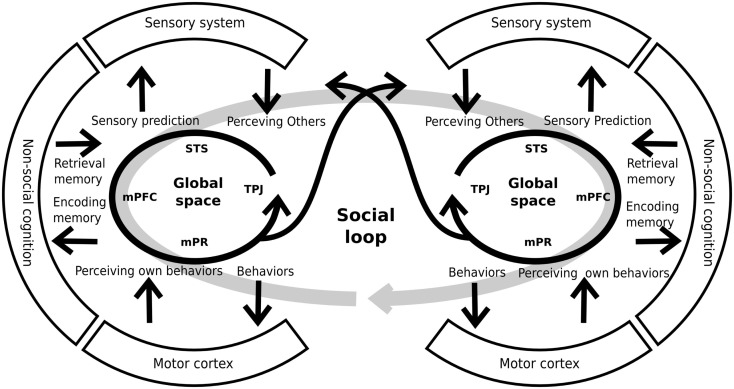
**Schematic representation of individual global spaces and the social loop**. Each person has a brain system generating global social representations. This system includes areas like the superior temporal sulcus (STS), the medial prefrontal cortex (mPFC), the medial parietal regions (mPR), and the temporo-parietal junction, among others. The global space has a strong relationship and interconnections with other systems (sensory, cognitive, and motor systems) that provide and receive information from them. By mean of these interconnections, the system generates adequate predictions and behaviors that allows the coupling of two, or more, individuals. For similar and complementary proposals see Decety et al. (2007), Hari and Kujala (2009), De Jaegher et al. (2010), Brunet-Gouet et al. (2011).

Interestingly, the constructs explained above have several shared areas and possibly shared neural systems. Moreover, the abnormalities taking place within any of these five constructs would concern directly the ability to represent others’ mental or emotional states. For this reason, other authors have proposed an integration of these constructs within a cohesive neurocognitive model (Decety et al., 2007; Brunet-Gouet et al., 2011). This proposal considers the existence of a “global” representation space that encompasses the cognitive systems which allow the online representation of things, persons, general semantic knowledge/associations, as well as more specific and complex past events. These representations are at the core of the information processing chain that allows us to make sense of our social environment and guide our daily social interactions. This online representation space can be subdivided into several subspaces, each of which deals with a specific type of social information and has a specific underlying brain system (Brunet-Gouet et al., 2011). Each of these brain systems is a specific interconnection among different brain areas of the global system rather than a modular brain system. We propose that each individual’s online representation space is coupled with that of other people. Thus, this coupling or engagement between two or more systems forms a global social system or social loop (see Figure 3), allowing us to interact with others, being generated by that interaction (Hari and Kujala, 2009). Crucially, this social loop has strong communication with both the sensory and the general cognitive systems. On the one hand, the social loop takes crucial information from the preceding systems (for example, memory of prior interactions) in order to generate adaptive behaviors. On the other hand, the social loop makes predictions on how the other agents might probably react to our behavior (for example, sensory predictions) and conveys them to the sensorial and the cognitive systems. Thus, this model incorporates the interactive nature of social cognition to the models which were proposed previously (see also De Jaegher et al., 2010). Interestingly, schizophrenic patients present alterations in both the subsidiary systems (e.g., sensory and cognitive systems) and the global social system. These impairments generate inadequate sensory predictions and behaviors that lead to a decoupling of the social loop and the poor social performance of schizophrenic patients.

While the skills which allow people to surf appropriately in their social world are not limited to understanding with others, they include both understanding and interacting with the social norms or cultural constraints (Vogeley and Roepstorff, 2009). Thus, both the perception of and the engagement with others are mediated by what people are expected to perceive and how people are expected to engage with others in their particular social and cultural context. For instance, there are cultural differences in the way how people make causal inferences in social contexts (Mason and Morris, 2010). Interestingly, cultural factors are in continuous dialectic interaction and exchange with individuals which constitute a specific culture. It follows that cultural factors and individual social practices exhibit a “looping effect” (Vogeley and Roepstorff, 2009). However, the study of the biological mechanisms of cultural social perception and engagement, and its implications in mental illnesses is a relatively unexplored area (Mehta et al., 2011).

## Conclusion

Humans are an intrinsically social and gregarious species, and virtually all of their actions (including their thoughts, desires, and feelings) are directed toward others or produced in response to others (Batson, 1990). Schizophrenic patients have problems with their interaction with other people and their integration in society. These problems seem to be due to specific impairments in social processing rather than consequences of general cognitive alterations. These impairments include at least three levels of processing, namely (1) the perception of social stimuli, (2) the understanding of other people’s mental states, and (3) the way in which people interact with other people and society. These steps involve different brain areas and different interactions or networks among the areas. In spite of the growing interest in the study of social cognition in schizophrenia, most of the research carried out in this area uses non-interactive paradigms, which do not capture the dynamic nature of the social phenomena involved. Hence, we propose a framework which does include the interactive nature of social skills. We believe that considering social skills as a result of dynamic, coupled systems is crucial to the better understanding of social cognition in both healthy people and psychopathologies, like schizophrenia. The use of these interactive paradigms, such as tasks based on game theory, allows us to develop clinical applications oriented to measuring and improving social skills in psychiatric populations.

## Conflict of Interest Statement

The authors declare that the research was conducted in the absence of any commercial or financial relationships that could be construed as a potential conflict of interest.

## Abbreviations

ACC, anterior cingulate cortex; AI, anterior insula; AMY, amygdala; BOLD, blood oxygen levels dependent; dlPFC, dorsolateral prefrontal cortex; EBA, extra-striate body area; EEG, electroencephalography; ERP, event-related potential; FFA, fusiform face area; fMRI, funtional magnetic resonance imaging; HTH, hypothalamus; MEG, magnetoencephalography; mPFC, medial prefrontal cortex; mPR, medial parietal regions; MSA, mental state attribution; MT, middle temporal (or visual area V5); OFC, orbitofrontal cortex; PC, precuneus. PCC, posterior cingulate cortex; STS, superior temporal sulcus; TPJ, temporo-parietal junction; VS, ventral striatum; vPMC, ventral premotor cortex.
